# Bio-Impedance Measurement Optimization for High-Resolution Carotid Pulse Sensing

**DOI:** 10.3390/s21051600

**Published:** 2021-02-25

**Authors:** Ting-Wei Wang, Hsiao-Wei Chu, Lin Chou, Yen-Ling Sung, Yuan-Ta Shih, Po-Chun Hsu, Hao-Min Cheng, Shien-Fong Lin

**Affiliations:** 1 Institute of Biomedical Engineering, College of Electrical and Computer Engineering, National Chiao Tung University, Hsinchu 30010, Taiwan; w756704@gmail.com (T.-W.W.); eric31506@gmail.com (H.-W.C.); choulin.arete09@nctu.edu.tw (L.C.); debbysong20000@gmail.com (Y.-L.S.); 2 Research and Development Department VI, Smart Healthcare BU, Leadtek Research Inc., New Taipei 23511, Taiwan; yt_shih@leadtek.com.tw (Y.-T.S.); pochun_hsu@leadtek.com.tw (P.-C.H.); 3 Center for Evidence-Based Medicine, Department of Medical Education, Taipei Veterans General Hospital, Taipei 11217, Taiwan; hmcheng@vghtpe.gov.tw; 4 Faculty of Medicine, National Yang-Ming University School of Medicine, Taipei 112304, Taiwan; 5 Institute of Public Health and Community Medicine Research Center, National Yang-Ming University, Taipei 112304, Taiwan

**Keywords:** bio-impedance measurement, carotid pulse sensing, cardiovascular monitoring, continuous blood pressure, hemodynamics, impedance plethysmography (IPG)

## Abstract

Continuous hemodynamic monitoring is important for long-term cardiovascular healthcare, especially in hypertension. The impedance plethysmography (IPG) based carotid pulse sensing is a non-invasive diagnosis technique for measuring pulse signals and further evaluating the arterial conditions of the patient such as continuous blood pressure (BP) monitoring. To reach the high-resolution IPG-based carotid pulse detection for cardiovascular applications, this study provides an optimized measurement parameter in response to obvious pulsation from the carotid artery. The influence of the frequency of excitation current, electrode cross-sectional area, electrode arrangements, and physiological site of carotid arteries on IPG measurement resolution was thoroughly investigated for optimized parameters. In this study, the IPG system was implemented and installed on the subject’s neck above the carotid artery to evaluate the measurement parameters. The measurement results within 6 subjects obtained the arterial impedance variation of 2137 mΩ using the optimized measurement conditions, including excitation frequency of 50 kHz, a smaller area of 2 cm^2^, electrode spacing of 4 cm and 1.7 cm for excitation and sensing functions, and location on the left side of the neck. The significance of this study demonstrates an optimized measurement methodology of IPG-based carotid pulse sensing that greatly improves the measurement quality in cardiovascular monitoring.

## 1. Introduction

Cardiovascular diseases (CVDs) are one of the main causes of death, according to the World Health Organization (WHO) statistics [1]. Thus, continuous hemodynamic monitoring is important for long-term cardiovascular diagnoses such as hypertension, thus further providing the treatment methodology. In clinical application, the intra-arterial catheter is a common method to monitor hemodynamic parameters such as blood pressure (BP) and pulse rate. However, the intra-arterial catheter is an invasive method that could induce adverse complications for the patient [2,3]. Recently, wearable non-invasive biomedical sensors were proposed to address the adverse events of the arterial cannula and provide real-time hemodynamic parameters monitoring such as pressure pulse wave and continuous BP measurement [4,5,6], as shown in Table 1.

One of the proposed approaches relies on the photoplethysmography (PPG) method. The optical technique-based device is based on light reflection or transmission to detect volumetric changes in the blood. The PPG function was widely applied in wearable devices [7,8] for heart rate [9] and BP [10] recording. However, the PPG approach could be applied only on the specific superficial artery such as the radial artery owing to the low penetration ability of light [11,12]. Other continuous hemodynamic monitoring techniques employ pressure sensors, including the capacitive pressure sensor and piezoelectric sensor. The capacitive pressure sensor measures arterial pulsation by detecting the distance change between two parallel plates and electrical capacitance [13,14]. The sandwich structure of the parallel plate capacitor has high stability, but the capacitive pressure sensors are typically characterized by low measurement sensitivities owing to the small variation of distance between the two electrodes [15]. For example, Kim et al. [16] presented a capacitive pressure sensor with the dielectric layer made of PDMS to enhance the measurement sensitivity of the sensor for BP monitoring. The piezoelectric sensor is based on the mechanical-electrical conversion by piezoelectric-sensitive material of polyvinylidene fluoride (PVDF) film, thus arterial pressure signals can be depicted by electrical signals recording [17,18]. Wang et al. [19] provided a piezoelectric-based system to extract pressure pulse waves from the radial artery and further calculate the beat-to-beat BP measurement. However, the pressure sensor required sufficient external pressure between skin and sensor to ensure the coupling condition would not change during the measurement. The main limitation of the pressure sensor is caused by discomfort from semi-occlusive measurement that could not be suitable for long-term monitoring.

The impedance plethysmography (IPG) technique utilized the principle of electrical impedance to depict the continuous bio-impedance waveform under small alternating current excitation [20,21]. Compared to PPG and pressure sensors, the IPG technique could address the light penetration issue of the PPG sensor and semi-occlusive measurement of the pressure sensor owing to the mechanism of the electrical impedance measurement [22]. Some studies utilized the IPG-based technique to apply in continuous BP measurement [22,23] and deep veins of the leg monitoring [24]. In our previous study [25], a single-channel IPG neck patch device was developed and installed on the subject’s neck above the carotid artery to perform cardiovascular monitoring, including the continuous BP and pulse rate.

This study mainly focused on investigating the influence of different measurement parameters on the measurement resolution of IPG-based carotid pulse sensing. The objective of this work is to determine the optimized condition that provides obvious physiological signals in response to arterial pulsation for improving the measurement quality in IPG-based cardiovascular applications. In this study, the different excitation frequencies, electrode area, electrode spacing, and physiological location were investigated to evaluate IPG measurement resolution.

The rest of this paper is organized as follows. Section 2 introduces the theoretical model of IPG-based carotid pulse sensing, IPG system design, bio-impedance calculation method for a carotid pulse, and experimental protocols. In Section 3, the results of different measurement parameters on IPG measurement resolution. In Section 4 and Section 5, the discussions and conclusions were drawn.

## 2. Methods

**Figure 1:**
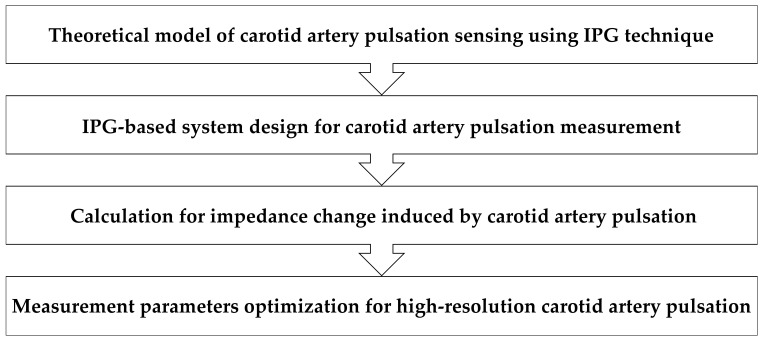
Flow chart of the IPG methodology for carotid pulse sensing.

The flow chart of the methodology of our study was presented in Figure 1. First, the theoretical model of IPG-based carotid pulse measurement was established. Second, the IPG device was designed for testing the concept. Third, the calculation method for impedance change induced by carotid artery pulsation was provided. Forth, the influence of different measurement parameters on IPG-based arterial pulsation was evaluated to determine the optimized measurement solution for carotid pulse sensing.

### 2.1. Theoretical Model of IPG-Based Carotid Artery Pulse Sensing

**Figure 2:**
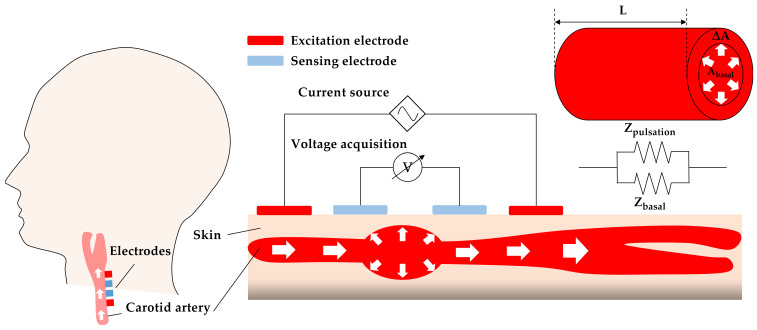
Schematic of IPG-based carotid pulse sensing and equivalent electrical model.

The IPG measurement is based on the principle of electrical impedance that uses two pair electrodes placed on the selected body segments. The excitation electrodes employ a small alternating current that drives into the human body. The sensing electrodes are performed to extract the change in arterial impedance under continuous alternating current excitation. In arterial pulsation sensing, the IPG technique can extract real-time arterial impedance induced by blood volume changes in the artery during the diastole and systole phases, as shown in Figure 2. The arterial impedance can be categorized into basel impedance (Z_basal_) and shunting impedance induced by pulsation (Z_pulsation_). Based on Ohm’s raw, Z_basal_ and Z_pulsation_ can be defined as Equations (1) and (2) by assuming that blood resistivity is consistent, arterial pulsation induced by blood volume change is uniform, and the driving currents are parallel with the carotid artery. In Equations (1) and (2), ρ, L, A_basal_, ΔA represent the blood resistivity, the measured length of the arterial segment, invariable basal cross-sectional area, and arterial cross-sectional area change of the artery:

(1) $$Z_{\mathrm{basal}}=\;\rho \;\frac{L}{A_{\mathrm{basal}}}$$

(2) $$Z_{\mathrm{pulsation}}=\;\rho \;\frac{L}{\Delta A}$$

The equivalent electrical model can regard as the Z_basal_ and Z_pulsation_ in parallel, according to Equation (3):

(3) $$Z(t)\;=\;{(\frac{1}{Z_{\mathrm{basal}}}\;+\;\frac{1}{Z_{\mathrm{pulsation}}})\;}^{-1}$$

The change in arterial impedance of carotid artery induced by volume change can be acquired as Equation (4).

(4) $${\Delta Z\;=\;(Z}_{\mathrm{basal}}{||\;Z}_{\mathrm{pulsation}}{)\;-\;Z}_{\mathrm{basal}}=\;-\frac{Z_{\mathrm{basal}}^{2}}{Z_{\mathrm{basal}}{\;+\;Z}_{\mathrm{pulsation}}}$$

Equation (5) can be simplified from Equation (4) by assuming that Z_basal_ is much smaller than Z_pulsation_ due to the larger cross-sectional area of A_basal_ than ΔA [26] under the consistent blood resistivity and measured length of the arterial segment:

(5) $$Z_{\mathrm{pulsation}\;}=\;-\frac{Z_{\mathrm{basal}}^{2}}{\Delta Z}$$

Thus, the arterial impedance change induced by blood volume change can be derived as Equation (6), according to Equations (2) and (5):

(6) $$\Delta Z\;=\;-\frac{Z_{\mathrm{basal}}^{2}}{\rho L}\Delta A\;$$

### 2.2. IPG-Based System Design for Carotid Pulse Measurement

**Figure 3:**
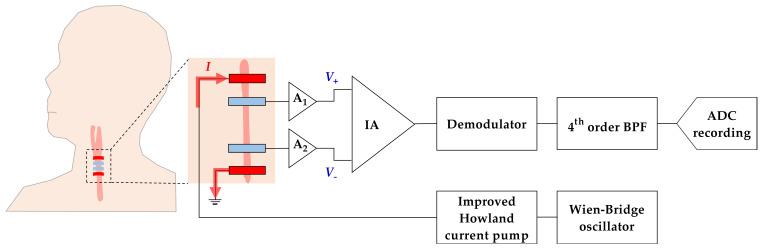
IPG system consists of two pairs of electrodes, two pre-amplifiers, instrumentation amplifier, demodulator, 4th order bandpass filter, Wien-Bridge oscillator, and improved Howland current pump.

Overall IPG system can be divided into three main parts, including electrodes, current source, and analog front-end (AFE), as shown in Figure 3. The wet-contact silver/silver chloride (Ag/AgCl) electrodes were utilized due to electrolytic gel with an excellent electrochemical characteristic [27,28]. The current source was implemented by a Wien-Bridge oscillator and an improved Howland current pump to produce an alternating current with an amplitude of 100 μA and frequency of 50 kHz, which allows the safety guideline in the human body [29,30,31]. To measure arterial impedance change induced by small arterial pulsation, the AFE was implemented for enlarging the IPG signals. The pre-amplifiers with ultra-high impedance 10^13^ Ω||1 pF (OPA124, Texas Instruments, Dallas, TX, USA) are connected to sensing electrodes for avoiding voltage drop. An instrumentation amplifier (IA) (AD8421, Analog Devices Inc., Norwood, MA, USA) was used to detect the difference in input voltage between V_+_ and V_−_. AD8421 provided a low input voltage noise of 3 nV/√Hz and high common-mode rejection ratio (CMRR) of 110 dB and a gain of 989 at 50 kHz to amplify IPG signals from the small arterial impedance change. To remove the 50 Hz carrier signals from alternating driving current, a demodulator IC (AD8310, Analog Devices Inc., Norwood, MA, USA) was utilized to extract the envelope signals induced by arterial pulsation. A four-order Butterworth bandpass filter was designed with a bandwidth from 0.3 Hz to 5 Hz to cover the range of pulse rate [32]. The analog IPG signals were transmitted into an analog-to-digital converter (ADC) for signal recording. The data acquisition module (myDAQ, National Instruments, Austin, TX, USA) provides a sampling rate of 500 S/s and 16-bit resolution per channel.

### 2.3. Calculation for Arterial Impedance Change

**Figure 4:**
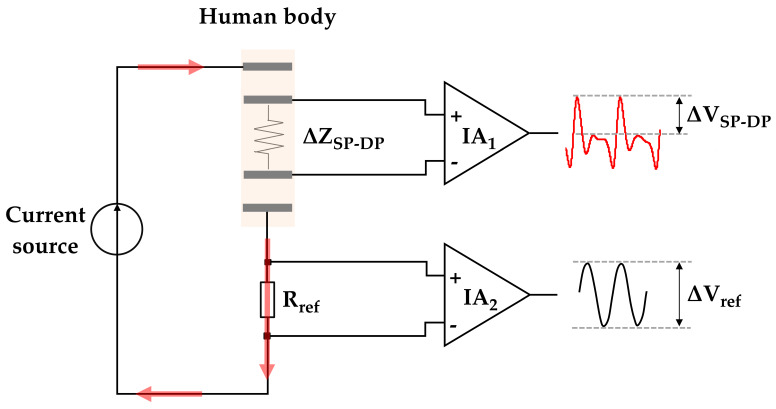
Schematic of the bio-impedance calculation method for the carotid pulse.

To measure the arterial impedance change during the systolic phase (SP) and diastolic phase (DP), the proportional method was utilized for the impedance measurement, as shown in Figure 4. The reference resistor of R_ref_ was connected to the IPG electrodes and current source in series. Two instrumentation amplifiers (IA_1_ and IA_2_) were used with the same components of OPA124. By assuming that the non-inverting and inverting terminals of IA can be neglected owing to ultra-high input impedance, thus ensuring the consistent current pass through the human body and reference resistor. Based on the Ohm’s raw, the voltage difference (ΔV_ref_) over the known resistor R_ref_ was used to calculate the impedance change in the artery between systolic and diastolic phases (ΔZ_SP-DP_) [21] through the measured voltage change in the artery (ΔV_SP-DP_), according to Equation (7):

(7) $${\Delta Z}_{\mathrm{SP}-\mathrm{DP}}{=\;R}_{\mathrm{ref}}\frac{{\Delta V}_{\mathrm{SP}-\mathrm{DP}}}{{\Delta V}_{\mathrm{ref}}}$$

### 2.4. Ethics Statement and Experimental Design

**Figure 5:**
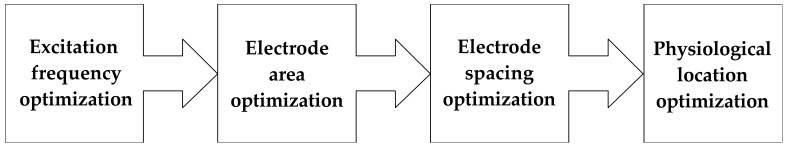
Flow chart of the step-by-step optimized procedure for measurement parameter.

The experiment was permitted by the Institutional Review Board of National Chiao Tung University (NCTU-REC-109-012E). A total of six healthy subjects (three males and three females) participated in the experiment, with the age of 21 ± 2 years, the height of 168 ± 9 cm, the weight of 70 ± 18 kg. The measurement experiments were performed on the neck of the participants by medical tape (Nexcare™ Micropore 17003, 3M™, St. Paul, MN, USA). In the installation of bio-impedance electrodes, two pieces of tape with the same length of 8 cm were used to attach the end of the electrodes to provide a consistent pressure during the bio-impedance measurement. All subjects agreed with the research ethics and were instructed to remain in a sitting position. The experimental flow chart was demonstrated to investigate the influence of measurement parameters (excitation frequency, electrode area, electrode spacing, and physiological location) on IPG resolution, as shown in Figure 5.

## 3. Experimental Results

### 3.1. Influence of Excitation Frequency on IPG Measurement Resolution

**Figure 6:**
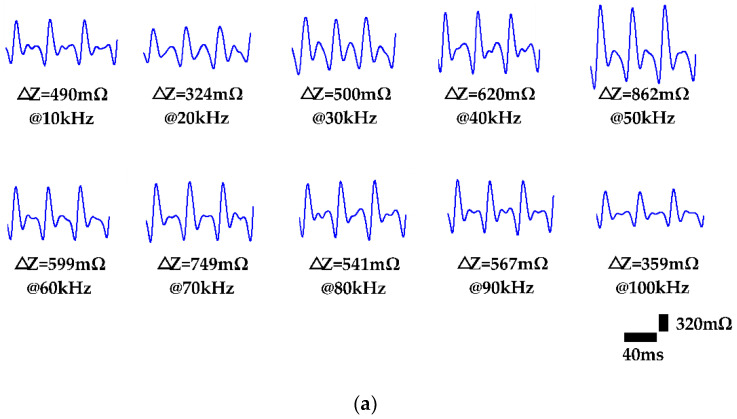

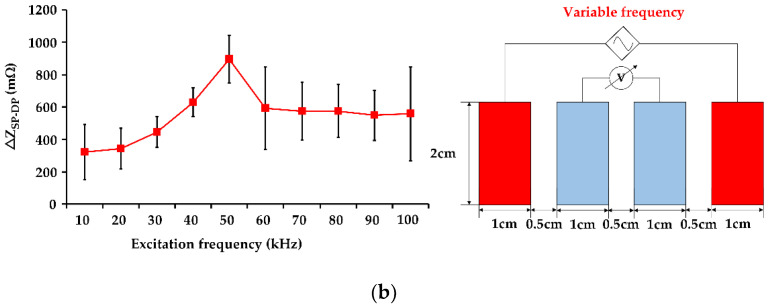
(**a**) IPG signals with excitation frequencies from 10 kHz to 100 kHz (Subject 1). (**b**) Statistical results for impedance variation in carotid pulse from 10 kHz to 100 kHz (n = 6).

The different frequencies of the driving current were performed (10 kHz–100 kHz) to evaluate the influence of excitation frequency on IPG measurement. In this assessment, the spacing of electrodes was an isometric arrangement with a spacing of 0.5 cm and the electrode area was 2 cm^2^. The electrode was placed above the right neck above the carotid artery of the subjects. Figure 6a demonstrates IPG morphology in different frequencies from 10 kHz to 100 kHz for subject 1. Figure 6b shows that a frequency of 50 kHz obtained a larger impedance difference (ΔZ_SP-DP_) of 895 mΩ than others.

### 3.2. Influence of Electrode Area on IPG Measurement Resolution

**Figure 7:**
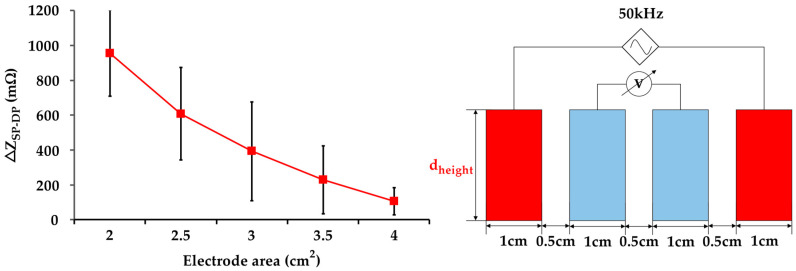
The influence of different electrode cross-sectional areas on IPG resolution measurement (n = 6).

To investigate the IPG measurement resolution from arterial pulsation, the different electrode area from 2 cm^2^ to 4 cm^2^ was provided. In the measurement experiment, the electrode spacing and current excitation frequency were 0.5 cm and 50 kHz. The experimental results demonstrate that the smaller electrode cross-sectional area of 2 cm^2^ provided higher resolution (ΔZ_SP-DP_ = 956 mΩ), as shown in Figure 7.

### 3.3. Influence of Electrode Spacing on IPG Measurement Resolution

**Figure 8:**
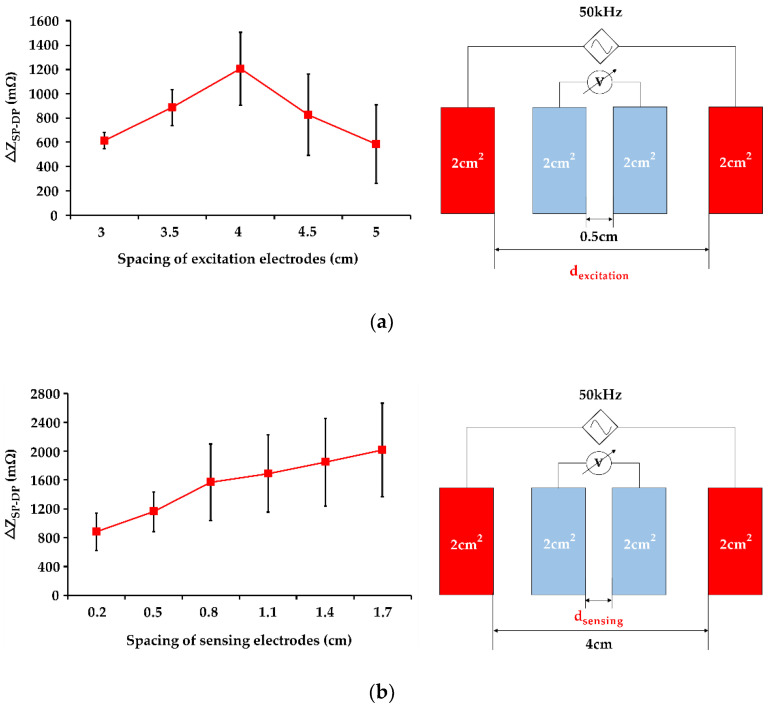
The influence of different (**a**) excitation spacing (from 3 cm to 5 cm) and (**b**) sensing spacing (from 0.2 cm to 1.7 cm) on IPG resolution measurement (n = 6).

The electrode placement also affects IPG measurement resolution [11,21]. To explore the influence of IPG electrode placement on the carotid artery, the spacing of excitation and sensing electrodes were evaluated for high-resolution IPG measurement. The excitation electrodes employ for conducting the driving current to pass through the artery regions. The spacing of the excitation electrode could affect the penetration depth of the current below the skin. To obtain the obvious impedance change in response to the arterial pulsation, the different spacing of excitation electrodes from 3 cm to 5 cm were evaluated. In this experiment, the spacing of the sensing electrode was determined by 0.5 cm. Figure 8a shows that a spacing of 4 cm obtained a higher IPG measurement resolution (ΔZ_SP-DP_ = 1206 mΩ). The sensing electrodes were utilized to detect the real-time arterial impedance under continuous and consistent current excitation. The variable spacing of sensing electrodes from 0.2 cm to 1.7 cm was provided to investigate the influence of sensing spacing on IPG measurement resolution. Figure 8b shows that the impedance difference enlarged as increasing the distance of sensing electrodes. A spacing of 1.7 cm reached the better IPG measurement resolution of ΔZ_SP-DP_ = 2015 mΩ.

### 3.4. Influence of Left and Right Side Carotid Artery on IPG Measurement Resolution

**Figure 9:**
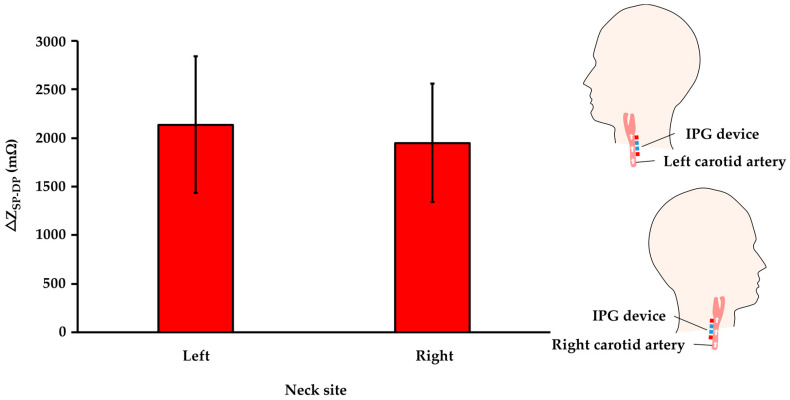
The comparison results of IPG measurement resolution between carotid arteries in the left and right sides (n = 6).

To investigate the influence of the carotid arteries in the left and right sides of the neck on IPG measurement, simultaneous IPG recording was performed in this study. Two same IPG systems were installed above the carotid arteries in right and left neck with consistent excitation frequency, electrode area, and electrode spacing. The comparison results show that the arterial impedance changes on the left side of the neck obtained a higher impedance difference of 2137 mΩ than the right side of 1949 mΩ, as shown in Figure 9.

## 4. Discussions

### 4.1. Significance in IPG-Base Carotid Pulse Sensing

The IPG-based carotid pulse sensing is useful for diagnosing hemodynamic parameters such as continuous BP. The IPG-based signals from the carotid artery deliver the pulse signals with the variation in arterial pressure and blood volume per heartbeat. The surficial carotid artery was selected for IPG physiological measurement location owing to palpable pressure pulse and the measurement location near the central aorta. The strong arterial pulsation from the carotid artery represents the larger blood volume changes in response to obvious impedance change by IPG sensing, according to Equation (6). Also, the measurement location of the carotid artery is near the central aorta. The central aortic pressure (CAP) reflected the true load imposed on the heart and large arteries rather than peripheral arteries [33]. However, the pressure signals of the central arteries must be extracted from the further transformation of alternative waveforms for non-invasive measurement techniques [26]. Compared to branchial and radial arteries, the waveform morphology of the carotid artery was similar to the aorta owing to the site near the aortic artery with lower pressure distortion [34]. Based on the physiological aspect of non-invasive hemodynamic monitoring, the IPG-based carotid artery has a high potential for cardiovascular applications.

### 4.2. Influence of Measurement Parameters on IPG Measurement Resolution

To this end, this study mainly researched the influence of measurement parameters on IPG-based carotid pulse measurement. In this study, the four approaches were provided for high-resolution IPG measurement of six participants, including (1) excitation frequency of the current source, (2) electrode area, (3) arrangements for excitation and sensing electrodes, and (4) physiological location of the carotid arteries. The Fricke-Morse model indicates that the excitation frequency of the current source affects biological impedance measurement [35,36]. The test frequencies from 10 kHz to 100 kHz were performed to evaluate the excitation frequencies on IPG measurement. The frequency of 50 kHz was the most effective excitation frequency in response to the obvious impedance change (ΔZ_SP-DP_) of 895 mΩ, as shown in Figure 6. Some studies indicated the frequency of 50 kHz was the most efficient excitation parameter for bio-impedance measurement, resulting in the high penetration depth through intracellular and extracellular fluids [37,38]. Besides, the electrode area was investigated for IPG-based carotid pulse sensing. The different electrode areas from 2 cm^2^ to 4 cm^2^ were tested to assess the IPG measurement resolution. Figure 7 shows that the IPG resolution was inversely correlated to the electrode area. The smaller electrode area of 2 cm^2^ obtained the higher ΔZ_SP-DP_ of 956 mΩ owing to voltage sensing at a specified location [39]. In contrast, the large area obtained the lower impedance change from carotid artery pulsation, which was attributed to the lower spatial resolution from IPG measurement under the large active area of electrodes. The electrode placing arrangement was also important for IPG measurement. Some studies also investigated the optimized spacing of electrodes for IPG-based radial artery applications [22,40]. This study focused on the carotid artery to evaluate the influence of electrode arrangement (excitation and sensing electrode pairs) on IPG measurement resolution. The optimized excitation electrode placing can greatly confine the electrical current pass through the region of the carotid artery and then obtain the high-resolution IPG waveform. Figure 8a demonstrates that a spacing of 4 cm obtained an obvious impedance variation of 1206 mΩ at testing excitation spacing from 3 cm to 5 cm. The spacing between two excitation electrodes would influence the measuring depth [41]. The narrow spacing within two excitation electrodes could pass through the superficial surface rather than the arterial region. The longer spacing within the excitation electrodes could decrease the current uniformity in the arterial region. For the consideration of sensing electrode spacing, the IPG measurement resolution was improved by increasing the spacing of sensing electrodes due to larger voltage differences under the consistent current source [22]. As shown in Figure 8b, the IPG measurement resolution was positively correlated to the spacing of sensing electrodes and a spacing of 1.7 cm obtained the maximum impedance difference of 2015 mΩ at testing sensing spacing from 0.2 cm to 1.7 cm. Most of all, this study investigated the influence of different sides of carotid arteries on IPG measurement resolution, as shown in Figure 9. The comparison results demonstrate the carotid artery on the left side obtained a higher ΔZ_SP-DP_ of 2137 mΩ than the right side of 1949 mΩ. The larger arterial pulse from the left common carotid artery (CCA) was attributed to a direct branch from the arch of the aorta. Compare to the left CCA, the right CCA is a branch of the brachiocephalic trunk [42,43]. Thus, the physiological asymmetry in the left and right carotid could induce the difference in impedance variation during the bio-impedance measurement.

### 4.3. Comparison with Related IPG Studies

We compared the IPG-based impedance variation results between previous studies and our work, as shown in Table 2. Based on Table 2, most studies [21,40,44] utilized the IPG-based wrist method to measure the IPG signals from the radial artery and investigated the influence of various measurement locations on impedance variation under the consistent excitation frequency and electrode area. Wang et al. [22] evaluated the influence of electrode spacing and excitation frequency on impedance variation in the radial artery. However, the measurement optimization of IPG-based carotid artery sensing has not been extensively investigated. Compared to the previous works, this study comprehensively evaluated the influence of the measurement parameters on IPG measurement resolution for carotid pulse sensing, resulting in an optimized result of 2137 mΩ. The robust performance of impedance variation could be attributed to the strong pulsation of the carotid artery in the physiological aspect than radial artery (Section 4.1). Importantly, the major contribution of our work is to optimize the measurement conditions of the excitation frequency, electrode area, electrode spacing, and physiological location for high-resolution IPG carotid pulse measurement (Section 4.2).

### 4.4. Limitations

Although this study demonstrates the influence of excitation frequency, electrode spacing, and physiological location on bio-impedance measurement, some limitations require further investigation in the future. First, the specific applied pressure on electrodes from medical tape needs to further evaluate for accurate bio-impedance measurement. Second, the mechanical stimulation of the carotid artery such as electrode installation could induce the carotid sinus reflexes, resulting in a small fall in heart rate and BP [45,46]. Third, recruit more subjects with hypertensive and hypotensive patients to further investigate the significant differences in ΔZ_SP-DP_ between different group conditions. Fourth, in Figure 7, the influence of lower electrode area (<2 cm^2^) on IPG measurement resolution needs to further investigate whether the ΔZ_SP-DP_ would become saturation until the d_height_ of testing electrodes approaches the width of the carotid artery.

## 5. Conclusions

This study investigated the influence of measurement parameters on IPG-based carotid pulse sensing. The excitation frequency, electrode cross-sectional area, electrode spacing, and physiological measurement site were evaluated for high-resolution IPG measurement in response to obvious carotid arterial pulsation. The measurement performance of six subjects obtained the obvious impedance variation of 2137 mΩ by excitation frequency of 50 kHz, electrode area of 2 cm^2^, excitation and sensing electrode spacing of 4 cm and 1.7 cm, and carotid artery on the left side. The main contribution of this study is to provide an optimized measurement condition of IPG-based carotid pulse sensing that improved the measurement quality in hemodynamic applications such as long-term BP monitoring.

## Figures and Tables

**Table 1:** Comparison for different pulse measurement techniques.

Technique	PPG	Pressure Sensor	IPG
Mechanism	light reflection or transmission	mechanical to electrical conversion	electrical impedance measurement
Advantage	wearable application	ruggedness	deep region detection
Challenge	superficial artery	semi-occlusive measurement	skin-electrode contact in dry electrode

**Table 2:** Performance comparison for IPG-based arterial pulse measurement.

Author	Artery	Participants	ΔZ_max_
Cho et al. [44]	radial artery	2 healthy subjects	131.1 mΩ
Huynh et al. [21]	radial artery	15 healthy subjects	325.8 mΩ
Huynh et al. [40]	radial artery	15 healthy subjects	67.1 mΩ
Wang et al. [22]	radial artery	30 healthy subjects	594 mΩ
Our work	carotid artery	6 healthy subjects	2137 mΩ

## Data Availability

The data presented in this study are available on request from the corresponding author.
